# Genomic and Enzymatic Results Show *Bacillus cellulosilyticus* Uses a Novel Set of LPXTA Carbohydrases to Hydrolyze Polysaccharides

**DOI:** 10.1371/journal.pone.0061131

**Published:** 2013-04-04

**Authors:** David Mead, Colleen Drinkwater, Phillip J. Brumm

**Affiliations:** 1 Lucigen Corporation, Middleton, Wisconsin, United States of America; 2 C5•6 Technologies, Middleton, Wisconsin, United States of America; 3 DOE Great Lakes Bioenergy Research Center, University of Wisconsin-Madison, Madison, Wisconsin, United States of America; University of Liverpool, United Kingdom

## Abstract

**Background:**

Alkaliphilic *Bacillus* species are intrinsically interesting due to the bioenergetic problems posed by growth at high pH and high salt. Three alkaline cellulases have been cloned, sequenced and expressed from *Bacillus cellulosilyticus* N-4 (Bcell) making it an excellent target for genomic sequencing and mining of biomass-degrading enzymes.

**Methodology/Principal Findings:**

The genome of Bcell is a single chromosome of 4.7 Mb with no plasmids present and three large phage insertions. The most unusual feature of the genome is the presence of 23 LPXTA membrane anchor proteins; 17 of these are annotated as involved in polysaccharide degradation. These two values are significantly higher than seen in any other *Bacillus* species. This high number of membrane anchor proteins is seen only in pathogenic Gram-positive organisms such as *Listeria monocytogenes* or *Staphylococcus aureus*. Bcell also possesses four sortase D subfamily 4 enzymes that incorporate LPXTA-bearing proteins into the cell wall; three of these are closely related to each other and unique to Bcell. Cell fractionation and enzymatic assay of Bcell cultures show that the majority of polysaccharide degradation is associated with the cell wall LPXTA-enzymes, an unusual feature in Gram-positive aerobes. Genomic analysis and growth studies both strongly argue against Bcell being a truly cellulolytic organism, in spite of its name. Preliminary results suggest that fungal mycelia may be the natural substrate for this organism.

**Conclusions/Significance:**

*Bacillus cellulosilyticus* N-4, in spite of its name, does not possess any of the genes necessary for crystalline cellulose degradation, demonstrating the risk of classifying microorganisms without the benefit of genomic analysis. Bcell is the first Gram-positive aerobic organism shown to use predominantly cell-bound, non-cellulosomal enzymes for polysaccharide degradation. The LPXTA-sortase system utilized by Bcell may have applications both in anchoring cellulases and other biomass-degrading enzymes to Bcell itself and in anchoring proteins other Gram-positive organisms.

## Introduction

Alkaliphilic *Bacillus* species are intrinsically interesting due to the problems posed by growth at high pH and high salt [1], [2], [3]. Proton motive force generation and ATP production is significantly different under these conditions than under neutral pH and low salt conditions [4]. The solutions to these problems that alkaliphilic *Bacillus* have developed are of continuing interest; for example, some species have modified ATP synthases that allow production at high pH [5], [6]. In others, a specific S-layer protein is linked to growth at high pH [7].

In addition to the interesting adaptive physiology of high pH microorganisms, the secreted enzymes produced by alkaliphilic *Bacillus* species are of interest for many reasons. The enzymes typically have exceptional alkaline tolerance as expected, but they are also resistant to oxidative environments, temperature, metals, surfactants, chelating agents, proteinases and high salt conditions. These properties make the enzymes excellent candidates for use in industrial applications. Many industrially relevant enzymes have been isolated from alkaliphilic species including xylanases from *B. halodurans* [8], [9], *Bacillus* sp. strain 41M-1[10], *B. firmus* [11], endoglucanases from *Bacillus sp.* [12], *B. agaradhaerens* [13], *B. halodurans* [14], amylases from *B. halodurans* [15], [16] *B. agaradhaerens* [17], *B. clarkii* 7364 [18], and proteases from *B. alcalophilus* [19], *B. halodurans* [20] and other isolates. Three alkaline cellulases have been cloned, sequenced and expressed from *Bacillus* N-4 [21], [22], [23], making it an excellent target for genomic mining of biomass-degrading enzymes. The taxonomy of *Bacillus* N-4, a soil organism that is both alkaliphilic and halophilic, has since been determined, and the organism has been given the species name, *Bacillus cellulosilyticus,* with N-4 (DSM 2522) being the type strain [24]. In spite of the interest in the alkaliphilic *Bacillus* species, only two of these organisms have had their genomes sequenced, *B. halodurans* [25] and *B. selenitireducens* MLS10 (unpublished, GenBank: CP001791.1, Gold ID: Gc01337). This work reports the genome sequence of *Bacillus cellulosilyticus* DSM 2522 and the novel enzymes found therein.

## Results

### General Genomics

The *Bacillus cellulosilyticus* DSM 2522 (Bcell) genome consists of a single, circular chromosome of 4,681,672 base pairs (GenBank: CP002394.1) with a GC content of 36.5%, lower than the 39.6% reported previously [24]. Gene prediction revealed 4,327 protein-encoding gene models and 61 pseudogenes (**Table 1**); 2876 (64.7%) had a function prediction and 1451 (32.7%) had none. A total of eight rRNA operons were found containing ten 5S rRNAs, ten 16S rDNAs, and ten 23S rRNAs. 81 tRNAs covering all 20 protein amino acids were also recovered (GenBank accession:CP002394), as well as 5 other RNA genes. The COGS Functional Groups Predictions (**Table 2**) show Bcell has the highest percentage of genes assigned to amino acid transport and metabolism (9.5%), carbohydrate transport and metabolism (8.7%), and inorganic ion transport and metabolism (6.8%).

**Table 1:** Nucleotide content and gene count levels of the Bcell genome.

Attribute	Genome (total)	
	Value	% of total^a^
Size (bp)	4681672	100.0
G+C content (bp)	1709838	36.5
Coding region (bp)	3802981	81.2
Total genes	4443	100.0
Pseudo genes	61	1.4
RNA genes	116	2.6
Protein-coding genes	4327	97.4
Genes with function prediction	2876	64.7
Genes without function prediction	1451	32.7
Genes in ortholog clusters	3745	84.3
Genes in paralog clusters	109	2.5
Genes assigned to COGs	3084	69.4
Genes with signal peptides	1253	28.2
Genes with transmembrane helices	1316	29.6

a ) The total is based on either the size of the genome in base pairs or the total number of protein coding genes in the annotated genome.

**Table 2:** COGS Functional Groups.

Information storage and processing				
Group	Bcell	Percent		Description
J	191	4.5		Translation, Ribosomal Structure and Biogenesis
K	280	6.6		Transcription
L	159	3.7		DNA Replication, Recombination and Repair
4	3	0.1		Chromatin structure and dynamics
**Cellular processes**				
D	136	3.2	Cell Division and Chromosome Partitioning	
V	129	3.0	Defense mechanisms	
T	217	5.1	Signal Transduction Mechanisms	
M	282	6.6	Cell Envelope Biogenesis, Outer Membrane	
N	110	2.6	Cell Motility and Secretion	
Z	0	0	Cytoskeleton	
U	57	1.3	Intracellular trafficking and secretion	
O	220	5.1	Posttranslational Modification, Protein Turnover, Chaperones	
**Metabolism**				
C	278	6.5	Energy production and Conversion	
G	373	8.7	Carbohydrate Transport and Metabolism	
E	407	9.5	Amino Acid Transport and Metabolism	
F	122	2.8	Nucleotide Transport and Metabolism	
H	242	5.7	Coenzyme Metabolism	
I	86	2.0	Lipid Metabolism	
P	290	6.8	Inorganic Ion Transport and Metabolism	
Q	151	3.5	Secondary metabolites biosynthesis, transport and catabolism	
**Poorly characterized**				
R	525	12.3	General Function Prediction Only	
S	291	6.8	Function Unknown	
Gene Count	4273			

Data for *B. cellulosilyticus* obtained from http://genome.ornl.gov/microbial/bcel/21jul10/fun.html

A distance tree based on finished genomes was constructed using Phylogenetic Distance Tree software to determine the relationship of Bcell to other *Bacillus* species. The tree was created using Blast2Tree (http://bioinfo.unice.fr/blast/) using the blastn alignment of 16S rDNA genes of >1200 nt on the NCBI database and dnadist and neighbor tools from the Phylip package (http://evolution.genetics.washington.edu/phylip/doc/). The completed tree was visualized using CLC Sequence Viewer 6 software.

**Figure 1:**
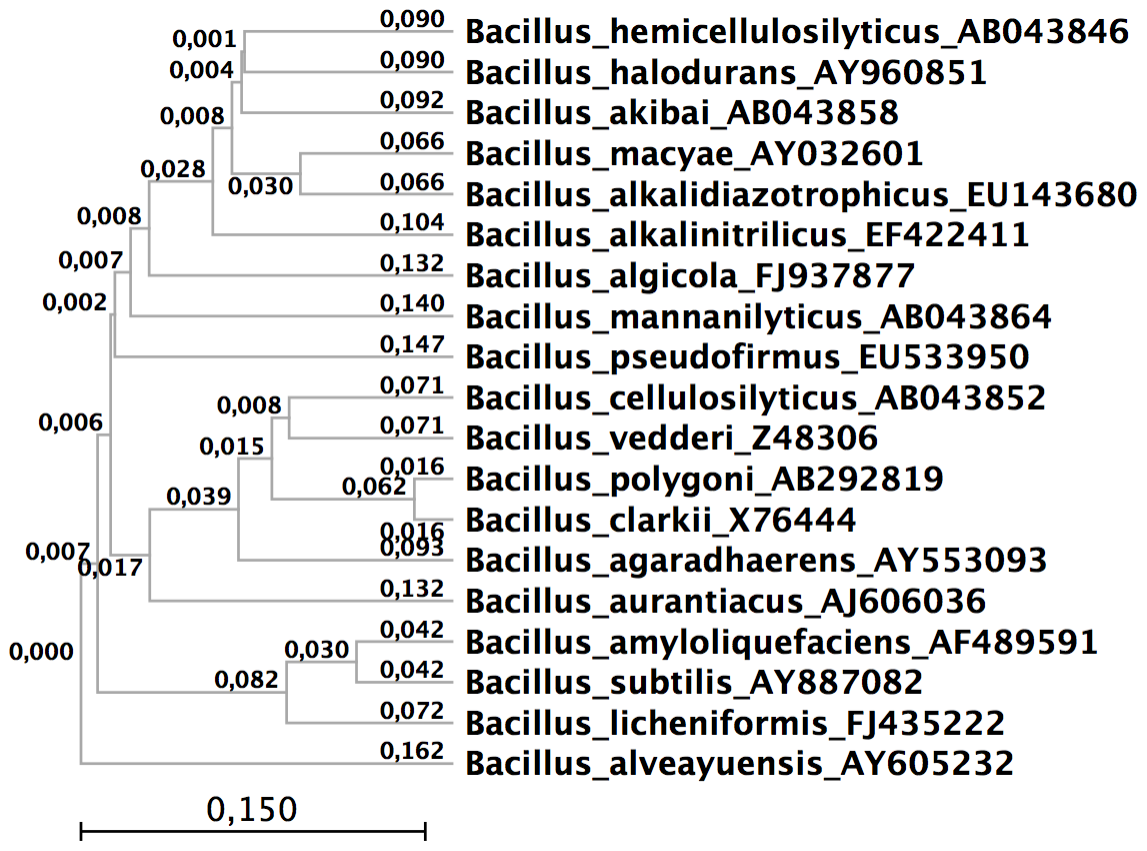
Phylogenetic tree highlighting the position of *Bacillus cellulosilyticus* N-4 DSM 2522 within the *Bacillales*. The strains and their corresponding NCBI accession numbers: *Bacillus hemicellulosilyticus* DSM 16731**^T^** AB043846; *Bacillus halodurans* XJU-2 AY960851; *Bacillus akibai* ATCC 43226**^T^** AB043858; *Bacillus macyae* DSM 16346**^T^** AY032601; *Bacillus alkalidiazotrophicus* UNIQEM U377**^T^** EU143680; *Bacillus alkalinitrilicus* UNIQEM U240**^T^** EF422411; *Bacillus algicola* LS7 FJ937877; *Bacillus mannanilyticus* DSM 16130**^T^** AB043864; *Bacillus pseudofirmus* SVB1 EU533950; *Bacillus cellulosilyticus* DSM 2522**^T^** AB043852; *Bacillus vedderi* DSM 9768**^T^** Z48306; *Bacillus polygoni* JCM 14604**^T^** AB292819; *Bacillus clarkia* DSM 8720**^T^** X76444; *Bacillus agaradhaerens* GSP78 AY553093; *Bacillus aurantiacus* B1-1 AJ606036; *Bacillus amyloliquefaciens* CMB01 AF489591; *Bacillus subtilis* AY887082; *Bacillus licheniformis* 11 FJ435222; and *Bacillus alveayuensis* KCTC 10634**^T^** AY605232.

The results (**Figure 1**) show that based on the 16S rDNA comparison, Bcell is most closely related to the alkalophile *B. vedderi*, an organism isolated from a bauxite-processing red mud tailing pond [26]. Bcell and *B. vedderi* are then related to two other alkalophiles, *B. polygoni* [27] and *B. clarkii* [28]. No genomic sequence information other than the 16S rDNA sequences has been published for these three organisms, making comparison with the Bcell genome impossible. Among the alkalophilic *Bacillus* species for which whole genome sequences are available, Bcell is distantly related to *B. selenitireducens* (Bsele) In spite of their apparent lack of relatedness by 16S rDNA comparison, it is of interest to see if these two alkalophilic *Bacillus* species are related on a whole-genome basis. The Bcell and Bsele genomes were annotated using the subsystem annotation system of the RAST server (Rapid Annotation using Subsytems Technology)[29]; the RAST server identified 3486 genes in Bsele and 4435 genes in Bcell. The annotated genomes were then analyzed to determine the number of homologous and unique proteins using SEED software[30]. Bcell and Bsele share 1084 proteins with >60% identity corresponding to approximately 30% of the genes in each genome. Shared proteins in Bcell and Bsele include ribosomal proteins, DNA and RNA synthetic enzymes, and enzymes of intermediary metabolism. Bcell possesses 1662 proteins without homologues (<10% identity) in Bsele. Of the Bcell unique protein annotations, 70% are to hypothetical proteins; among the identified proteins, approximately 70 proteins are involved in spore formation or germination and approximately 30 are involved in polysaccharide degradation, metabolism, or synthesis. Bsele possesses 1065 proteins without homologues in Bcell Of the Bsele unique protein annotations, 60% are to hypothetical proteins; among the proteins with identified function, a significant number code for proteins involved in anaerobic metabolism and phage proteins. To confirm this analysis, the Bsele protein orthologs of annotated Bcell proteins were determined using the IMG Genome Gene Best Homologs function (http://img.jgi.doe.gov/cgi-bin/w/main.cgi?section=GenomeGeneOrtholog). Of the Bcell proteins predicted by this annotation software, again, only 30% have orthologs in Bsele with ≥60% identity.

### Genome Features

#### Phage

Three phage integration events were identified by BacMap software http://bacmap.wishartlab.com/. The integration events resulted in insertions of phage DNA at 2,385,688 to 2,326,716 (41,209 bp); 2,902,461 to 2,946,144 (43,684 bp); and 3,312,514 to 3,323,401 (10,888 bp). The first two inserts are identified by the software as intact prophage, while the third is identified as an incomplete prophage. A screen of the genome revealed that Bcell has no CRISPR sequences, indicating a low level of defense against phage attack.

#### Salt and Alkaline Tolerance

Bcell is reported to grow in 12% but not 15% NaCl and optimally at pH values of 9 to 10 [24] and we have confirmed these observations with the strain. Genes for salt and alkaline tolerance include an operon coding for MrpA through MrpG (Bcell_1564 through Bcell_1558) as well as a gene encoding for an NhaC antiporter (Bcell_2073). Both of these have been linked to the alkaline and salt tolerance of other alkaliphilic *Bacillus* species (reviewed in [2]). The benefits of these features are not obvious if Bcell is truly a soil organism; Bcell's preferred habitat may actually be a saline and alkaline aqueous environment. Similar halophilic organisms have been isolated from soil around Tokyo, Japan [31]; the authors believed that these organisms originated from dust storms carrying material from highly saline environments, such as salt lakes in Mongolia or salterns in Korea.

#### Carbohydrate Metabolism Pathways

Bcell is reported to produce acid but not gas from a number of substrates including fructose, glucose, mannose, and *alpha* and *beta*-linked glucosides such as arbutin, cellobiose, salicin, maltose, maltotriose, sucrose, and lactose [24]. We performed a metabolic reconstruction analysis using the computer program PRIAM [32]which generates KEGG [33] maps. Metabolic pathway reconstruction of Bcell shows a complete set of enzymes for the hydrolysis of *alpha* and *beta*-linked glucosides to glucose and a full *Embden-Meyerhof-Parnas* pathway with enzymes for production of acetate and lactate. The metabolic reconstruction also shows a complete Krebbs cycle and oxidative phosphorylation complexes. Metabolism of complex carbohydrates will be discussed below.

#### Amino acid and Coenzyme Pathways

Metabolic pathway reconstruction of Bcell shows a complete set of enzymes for the synthesis of all twenty amino acids. The reconstruction indicates that Bcell has complete pathways for riboflavin, nicotinate, folate, pantothenate, and heme, but cannot synthesize thiamin, biotin, or pyridoxine.

#### Antibiotic Production and Resistance

Being a member of the *Bacillales,* who are well known for their ability to produce antibiotics [34] one would expect that Bcell might possess r antibiotic production genes.. *B. halodurans*, another alkaliphilic halophile, produces a two-peptide antibiotic, haloduricin [35], encoded by an operon containing six genes (BH0450–BH0455). BLAST comparison of these six genes from *B. halodurans* against the genome of Bcell gave no matches, indicating Bcell does not possess the ability to make this class of antibiotic. A KEGG Metabolic Pathways with PRIAM search of the complete genome revealed no genes for production of type I or type II polyketide backbones, nonribosomal peptide structures, siderophore group nonribosomal peptides, streptomycin, vancomycin, tetracycline, or novobiocin.

While Bcell does not produce identifiable antibiotics, it does possess several mechanisms for detoxifying a number of antibiotics. In culture, Bcell grows in the presence of 12 µg/ml of tetracycline without a lag and to an absorbance comparable to the antibiotic-free controls. Tetracycline resistance in *Bacillus* species typically utilizes efflux proteins coded for by EmrB/QacA family transporter genes. Bcell possesses four annotated EmrB/QacA family transporter genes, Bcell_0365, Bcell_1274, Bcell_2279, and Bcell_2272, any or all of which may be responsible for tetracycline efflux.

Bcell grows in the presence of 50 µg/ml of ampicillin and carbenicillin after a 24 hr lag phase to an absorbance comparable to the antibiotic-free controls. Subcultures of cells grown in media containing either antibiotic have no lag phase. Resistance to ampicillin and carbenicillin typically proceeds via *beta*-lactamase inactivation of the antibiotic. Bcell possesses eight annotated *beta*-lactamase genes (Bcell_0642, Bcell_0725, Bcell_0931, Bcell_1188, Bcell_2001, Bcell_2765, and Bcell_3823 and Bcell_3957) as well as thirteen annotated *beta*-lactamase domain-containing protein genes.

Bcell grows in the presence of 30 µg/ml of kanamycin after a 24 hr lag phase to an absorbance comparable to the antibiotic-free controls. Subcultures of cells grown in media containing kanamycin have no lag phase. Kanamycin resistance can arise via one of three mechanisms, ribosome alteration, altered permeability, or inactivation of the antibiotic. To determine if Bcell possessed one of the enzyme families responsible for kanomycin inactivation, the Bcell genome was checked for homologs to each of the three types of enzymes. BLAST comparison of the Bcell genome against *Bacillus* aminoglycoside 3′-phosphotransferase (P00553) gave no hits, indicating Bcell does not inactivate kanomycin by attaching a nucleotidyl group from nucleoside triphosphates such as ATP to the 4′-hydroxyl group of the antbiotic. Similarly, BLAST comparison of the Bcell genome against a *Streptomyces* kanamycin 6-acetyl transferase (Q65CD7) gave no hits, indicating Bcell does not inactivate kanomycin by attaching an acetyl group to the antibiotic. BLAST comparison of the Bcell genome against a *Streptomyces griseus* streptomycin 6-kinase (P08077) showed strong homology to Bcell_0299. BLAST comparison of Bcell-0299 to the nonredundant protein database showed homology to a number of identified streptomycin 6-phosphotransferase molecules suggesting that Bcell_0299 is responsible for kanomycin resistance in Bcell.

#### Flagellar and PilinBiosynthesis

The Bcell genome contains 34 annotated flagella-related proteins; 24 are located in an operon from Bcell_2483 through Bcell_2506 (2684873 through 2704278), three are located at Bcell_1751 through Bcell_1753 (1943374 through 1945855), three at Bcell_3600 through Bcell_3602 (3858394 through 3860973) and four at Bcell_3622 through Bcell_3625 (3881849 through 3885911). Bcell appears to contain no genes for pilin or pilin assembly.

#### Sortases and Membrane Anchor Proteins

Sortases are membrane transpeptidases found in gram-positive bacteria that anchor surface proteins to peptidoglycans of the bacterial cell wall envelope. These enzymes catalyze a transpeptidation reaction in which the surface protein substrate is cleaved and covalently linked to peptidoglycan for display on the bacterial surface (recently reviewed in [36]). Sortases are grouped into four different classes (sortase A, B, C, and D) and two subfamilies (sortase D subfamily 4 and 5) based on sequence homology and cleavage site preferences [37], [38]. Sortase A molecules recognize LPXTG sequences and anchor as many as twelve different substrates. Sortase B molecules recognize NPXTN sequences and anchor proteins involved in iron acquisition [39]. Sortase C recognize LPXTG sequences and are involved in pilin polymerization [40]. Sortase D subfamily 4 molecules recognize LPXTA sequences while the subfamily 5 molecules recognize LAXTG sequences. Their substrates are predicted to be predominantly enzymes such as 5′-nucleotidases, glycoside hydrolases, and proteases [37], though this has not been confirmed. Sortase D family members are often found adjacent to the postulated substrate for the sortase.

The genome of Bcell reveals the presence of four annotated sortase genes, Bcell_0651, Bcell_1334, Bcell_3485, and Bcell_3654 and BLAST analysis indicates all four are members of the sortase D subfamily 4. There are no members of sortases families A, B, or C. The Bcell sortases group into two families. Bcell_0651 is adjacent to an annotated LPXTA protein, Bcell_0652. Bcell_1334, Bcell_3485, and Bcell_3654 are not adjacent to any proteins containing the LPXTA domain. Bcell_0651shows low homology to the other three sortase genes of Bcell, but high homology (>70% identity) to sortases identified in *B. megaterium*, *B. halodurans*, and *B. licheniformis*. Bcell_1334, Bcell_3485, and Bcell_3654 show high homology to each other, but low homology (≤40%) to sortases of other *Bacillus* species.

The Bcell genome contains 27 annotated proteins with a membrane anchor domain (TIGR01167 domain). All 27 contain the conserved LPXTA sequence (**Table 3**); there are no proteins containing other LPXTG sequences recognized by sortase families A, B, or C. The next two amino acids in the LPXTA sequence appear to be highly conserved also; in 24 of the 27 proteins the sequence is one of the following: LPXTATN, LPXTATS, or LPXTATT. The remaining three proteins have LPXTATH (Bcell_0652), LPXTATR (Bcell_0381), or LPXTATD (Bcell_3788) sequences. Preceding the LPXTA sequence is a negatively charged, unorganized linker region connecting the membrane anchor to the highly organized functional domains. Following the LPXTA sequence is a membrane-spanning hydrophobic stretch of amino acids and finally a positively-charged C-terminal tail.

**Table 3:** Anchor Domain Sequences of LPXTA-motif Proteins

Gene	Blast Prediction	LPXTA Anchor Domain
0305	hypothetical protein	**LPTTAT**NTYNYLLIGIIALIAGIAFFLW*SRKKKMKIDS*
0381	internalin-type protein	**LPLTAT**RTYQFLLAGIIMLVGGSCIYVFY*RRRNIMKT*
0481	chitinase	**LPETAT**TMYNWLLIGAMLLIVGFTFLFI*SRKRKLQTTD*
0513	5′ nucleotidase	**LPDTAT**DVYQWLLAGLLLIFLGISLTLI*RNRHKTV*
0524	β-glucanase	**LPSTAT**NAYNYLLVGMLLLVIGGVSFFI*RRKQLG*
0541	xylanase	**LPRTAT**NNYNMIALGSLLLLLGTTILIVF*RRKNKVTIRE*
0652	hypothetical protein	**LPNTAT**HSLLNILLGFFASGAGLYFF*RKVKVL*
0683	β-glucanase	**LPNTAT**NLFNYLVIGMLLLIIGAVTFF*TRKKHVFNN*
0690	1,3 β-glucanase	**LPNTAT**NVYNLLIVGFLLLLLGSIIAY*QKRKSFTV*
1033	β-xylosidase	**LPDTAT**SIYNWLFVGISFILLGYLLIII*KRKSKGLYMN*
1191	cellulase	**LPDTAT**NLYNYLLIGLLMIIVGGTVTIF*SRKRKVVDM*
1280	1,3 β-glucanase	**LPDTAT**NAYNYLMVGMLLLLIGGVTFFI*RRKQLG*
1368	β-mannosidase	L**PDTAT**NMFNYFLLGLVLLMIGGMTFVF*SRKNRMIE*
2860	surface/cell adhesion protein	**LPDTAT**SNYHVLALGALFFLIGTVIFFT*QRRKVLN*
2962	arabinogalactan endo-1, 4-β-galactosidase	**LPDTAT**SKFNLFMLGMLLLVIGTTVFFL*SRRKQLRL*
3370	endoglucanase C	**LPDTAT**NTFNFLIIGALLLMSGVAFFVI*QRRKVATN*
3371	endoglucanase C	**LPNTAT**NMFNFLMVGLMLLAIGIVTFVI*KRKNVAIK*
3391	endoglucanase D	**LPDTAT**STFNYLLLGTVLFMLGTAIFLL*QRRKLLAK*
3504	O-glycoside hydrolase	**LPDTAT**NMYSSLLIGFILLSIGGVIFFV*TKKRYRAE*
3729	1,3 β-glucanase	**LPNTAT**NLFNYLVIGMLLLIIGAVTFF*TRKKQVFNN*
3731	glucose dehydrogenase	**LPSTAT**SMYNYLFIGFFILIIGCSLYFL*SKKGKRQEVNR*
3787	pullulanase	**LPDTAT**NTYTVMLMGLVLLLLGGATLFVI*RKKSVKEQSEL*
3788	α-amylase	**LPDTST**SMYNVLLIGSMLFLVGAGLYLF*NRKKALVK*
3820	nuclease	**LPATAT**NMYNMILIGLLLMTIGYTTITI*RKKQLS*
3829	GLUG domain protein	**LPSTAT**TTYNILLIGLILLIIGGVFL*KRRLLKVED*
3857	hypothetical protein	**LPNTAT**NVYNQLLLGAILILGGGVVLFGS*RKIKRRQRPYVIS*
3995	hypothetical protein	**LPSTAT**NNYNMLLLGTVLTIMGVISLYY*RKKRTA*

**Bold**: **LPXTAT domain**; underline: membrane-spanning region*; italics: intracellular positive tail*

The uniqueness of these LPXTAT-anchor proteins can be seen by comparing the number of LPXTAT-anchor proteins in Bcell to the number of total membrane anchor proteins in other *Bacillus* species. Based on a search for proteins containing the TIGR01167 domain, Bcell possesses 27, while the next highest, *B. halodurans*, possesses only six. *B. licheniformis* possesses three, and other *Bacillus* species possess only one or two. The value of 27 for Bcell is closer to that of pathogenic Gram-positive organisms such as *Listeria monocytogenes* strains (31 to 39 LPXTG proteins) or *Staphylococcus aureus* strains (10 to 18 LPXTG proteins).

Of the 27 proteins containing the conserved LPXTAT sequence, 17 are annotated as being involved in polysaccharide metabolism. Of the remaining nine, two are annotated as nucleic acid-degrading enzymes (Bcell_0513 and Bcell_3820), and are possibly involved in uptake of ssDNA. Three of the remaining seven are annotated as proteins potentially involved in cell adhesion (Bcell_0381, Bcell_2860 and Bcell_3829), and the remaining four are annotated as hypothetical proteins. Bcell possesses no annotated LPXTA proteases or lipases.

#### Polysaccharide Degradation by Bcell

Based on the genome annotation, polysaccharide degradation by Bcell appears to utilize a unique combination of LPXTA membrane anchor proteins combined with soluble extracellular enzymes and intracellular enzymes; this combination of enzymes is not found in any other identified *Bacillus* species. The enzymology of degradation of individual families of carbohydrates will be discussed separately. In all following discussions, BLAST [41] was utilized for functional annotation, SignalP for signal peptide determination [42], and CAZy for glycoside hydrolase (GH) and carbohydrate binding modules[43] (CBM).

Enzymes potentially involved in glucan degradation are shown in **Table 4**. The organism possesses four glycoside hydrolase family 5 [43] (GH5) and one GH9 secreted endoglucanases/cellulases; all six genes have annotated signal peptides, indicating secretion. Five of these six enzymes possess LPXTA anchors. All five GH5 family members possess carbohydrate binding modules[43] (CBM); the single GH9 family member does not. The CBM families present, CBM5, CBM17, and CBM46 are not associated with crystalline cellulose degradation. CBM5 primarily binds to chitin, CBM17 to amorphous cellulose and cellulose derivatives, and too little is known about CBM46-containing GH5 family members to assign a clear function. CBM1, CBM2, or CBM3 family members are not associated with any of these proteins, indicating that these enzymes are not involved in crystalline cellulose degradation. This confirms observations that cloned Bcell cellulases had strong activity on carboxymethyl cellulose, but low activity on Avicel crystalline cellulose [44]. Potential alternative substrates for GH5 family members include β-1,3-1,4-glucans, mannans and xyloglucans; any one or more of these may be the natural substrate for these enzymes. Bcell possesses eight β-glucanase genes, five GH16 (lichenases, 1,3-1,4-β-D-glucanases) and three GH81 (curdlanases, 1,3-β-D-glucanases). All eight genes have annotated signal peptides, indicating secretion; four of these eight β-glucanases have LPXTA anchors. Six of the eight β-glucanases have CBM modules from families 4, 6, or 56, all consistent with β-glucan degradation. CBM4 and CBM6 bind to a variety of carbohydrate polymers including β-1,4-xylan β-1,3-glucan, β-1,3-1,4-glucan, and β-1,4-glucan. Binding of CBM 56 has been demonstrated with β-1,3-glucan [45]. Bcell has genes coding for three intracellular β-glucosidases as well as one apparently secreted β-glucosidase gene, Bcell_3759. The organism also possesses genes coding for two cellobiose phosphorylases. This is highly unusual in an organism that does not possess “true” cellulases or degrade cellulose; the function of the cellobiose phosphorylases may be to degrade cellobiose generated in the degradation of β-1,3-1,4-glucan or xyloglucan.

**Table 4:** Annotated Glucan-degrading Enzymes.

Protein	Blast Prediction	Signal Peptide	GH	CBM	LPXTA Anchor
Bcell_0524	β-glucanase	yes	GH16GH16	CBM4 CBM4 CBM4	yes
Bcell_0683	β-glucanase	yes	GH16	CBM4 CBM4 CBM4 CBM4	yes
Bcell_1191	cellulase	yes	GH5	CBM46	yes
Bcell_1280	1,3 β-glucanase	yes	GH81	CBM4	yes
Bcell_3370	endoglucanase C	yes	GH5	CBM17/28 CBM17/28	yes
Bcell_3371	endoglucanase C	yes	GH5	CBM17/28 CBM17/28	yes
Bcell_3391	endoglucanase D	yes	GH9		yes
Bcell_3729	1,3 β-glucanase	yes	GH81	CBM56 CBM56	yes
					
Bcell_0437	endoglucanase B	yes	GH5	CBM5	no
Bcell_0438	endoglucanase A	yes	GH5	CBM5	no
Bcell_0690	β-glucanase	yes	GH16	CBM4 CBM4 CBM4	no
Bcell_1430	β-glucanase	yes	GH16		no
Bcell_2363	β-glucanase	yes	GH16	CBM6	no
Bcell_4185	1,3 β-glucanase	yes	GH81	CBM6 CBM6 CBM56	no
Bcell_3759	β-glucosidase	yes	GH3		no
					
Bcell_0282	β-glucosidase	no	GH1		no
Bcell_0705	β-glucosidase	no	GH3		no
Bcell_4264	β-glucosidase	no	GH3		no
Bcell_0478	cellobiose phosphorylase	no	GH94		no
Bcell_2329	cellobiose phosphorylase	no	GH94		no

Enzymes potentially involved in xylan degradation are shown in **Table 5**. The organism possesses two annotated GH10 xylanases, one intracellular, and one secreted with an LPXTA anchor. This is similar to the case in many *Bacillus* species, which possess both a secreted, non-LPXTG xylanase and an intracellular xylanase. The organism also has an annotated GH30 secreted xylanase and GH8 intracellular annotated xylanase. Completing the enzymes necessary for xylan degradation, Bcell has three secreted β-xylosidases (GH39 and GH43), one with an LPXTA anchor, as well as four intracellular annotated β-xylosidases (GH39 and GH43). Two additional intracellular enzymes may be involved in xylooligosaccharide degradation, a GH67 α-glucuronidase and a GH30 O-glycoside hydrolase. Two of the secreted enzymes possess CBM modules. The CBM families present, CBM9 and CBM22 are involved in xylan binding, while CBM35 binds to a number of substrates including xylan, mannan, and galactan. Bcell also possesses a total of eleven Carbohydrate Esterase[43] (CE) family members (**Table 6**), of which 3 CE4 members are annotated as xylan deacetylases; all three have signal peptides and may be involved in extracellular degradation of xylan.

**Table 5:** Annotated Xylan-degrading Enzymes.

Protein	Blast Prediction	Signal Peptide	GH	CBM	LPXTA Anchor
Bcell_0541	xylanase	yes	GH10	CBM9 CBM22 CBM22 CBM22	yes
Bcell_1033	β-xylosidase	yes	GH43		yes
					
Bcell_0821	xylanase	yes	GH30		no
Bcell_1039	β-xylosidase	yes	GH43		no
Bcell_1102	β-xylosidase	yes	GH39	CBM35, CBM35, CBM35	no
Bcell_0537	xylanase	no	GH8		no
Bcell_0547	xylanase	no	GH10		no
Bcell_0538	β-xylosidase	no	GH39		no
Bcell_0385	β-xylosidase	no	GH43		no
Bcell_0554	β-xylosidase	no	GH43		no
Bcell_1042	β-xylosidase	no	GH43		no
Bcell_0548	α-glucuronidase	no	GH67		no
Bcell_0689	O-glycoside hydrolase	no	GH30		no
Bcell_3505	O-glycoside hydrolase	no	GH30		no

**Table 6:** Carbohydrate Esterases.

Protein	Blast Prediction	Signal Peptide	CE Family	LPXTAanchor
Bcell_0165	xylan deacetylase	yes	CE4	no
Bcell_0810	xylan deacetylase	yes	CE4	no
Bcell_1432	xylan deacetylase	yes	CE4	no
Bcell_2338	deacetylase	yes	CE4	no
Bcell_2449	sporulation deacetylase	yes	CE4	no
Bcell_2662	deacetylase	yes	CE4	no
Bcell_4035	deacetylase	yes	CE4	no
Bcell_0236	xylan deacetylase	no	CE4	no
Bcell_3697	NAGphosphate-deacetylase	no	CE9	no
Bcell_1899	deacetylase	no	CE14	no
Bcell_4151	LmbE family protein	no	CE14	no

Enzymes potentially involved in starch degradation are shown in **Table 7**. The organism possesses three annotated GH13 amylases, two secreted with an LPXTA anchor and one remains intracellular. The secreted enzymes both have CBM41 modules; this module is reported to bind to amylose, amylopectin and pullulan, consistent with an amylolytic function. Starch degradation is completed intracellularly by two GH31 α-glucosidases, one with a CBM56.

**Table 7:** Annotated Starch-degrading Enzymes.

Protein	Blast Prediction	Signal Peptide	GH	CBM	LPXTAanchor
Bcell_3787	pullulanase	yes	GH13	CBM41CBM41	yes
Bcell_3788	α-amylase	yes	GH13	CBM41 CBM41	yes
Bcell_0491	α-amylase	no	GH13		no
Bcell_1202	α-glucosidase	no	GH31		no
Bcell_3789	α-glucosidase	no	GH31		no

Enzymes potentially involved in galactan degradation are shown in **Table 8**. The organism possesses two potential secreted galactan-degrading enzymes, a GH30 and a GH53, both with an LPXTA anchor. The secreted enzymes both also have CBM61 modules that are reported to bind to galactans; in addition, one has a CBM47 and one has a CBM6. Galactan degradation is completed intracellularly by two GH4 α-galactosidases, two GH36 α-galactosidases, four GH2 β-galactosidases and two GH42 β-galactosidases.

**Table 8:** Annotated Galactan-degrading Enzymes.

Protein	Blast Prediction	Signal Peptide	GH	CBM	LPXTAanchor
Bcell_2962	Arabinogalactan endo-1 4-β-galactosidase	yes	GH53	CBM47 CBM61	yes
Bcell_3504	O-glycoside hydrolase	yes	GH30	CBM6 CBM61 CBM61	yes
Bcell_1453	α-galactosidase	no	GH4		no
Bcell_2800	α-galactosidase	no	GH4		no
Bcell_1099	α-galactosidase	no	GH36		no
Bcell_1103	α-galactosidase	no	GH36		no
Bcell_0281	β-galactosidase	no	GH2		no
Bcell_0684	β-galactosidase	no	GH2		no
Bcell_1041	β-galactosidase	no	GH2		no
Bcell_0281	β-galactosidase	no	GH2		no
Bcell_1192	β-galactosidase	no	GH42		no
Bcell_2963	β-galactosidase	no	GH42		no

Enzymes potentially involved in chitin degradation are shown in **Table 9**. Bcell possesses one secreted GH18 chitin-degrading enzyme, with a LPXTA anchor and no CBM module. Chitin degradation is completed intracellularly by two GH18 chitinases with CBM50 modules and a GH20 chitobiase with no CBM module. The organism possesses one potential secreted mannan-degrading enzyme, a GH26 family member. The enzyme possesses a LPXTA anchor and CBM23, CBM28 and CBM59 modules. Binding of CBM23 and CBM59 to mannan has been demonstrated, while CBM28 selectivity is not well-characterized. Surprisingly, Bcell possesses no CAZy [43] pectate lyase family members (PL) or GH28 family members (pectinases), indicating the organism is unable to degrade pectin and related polysaccharides such as rhamnogalacturonans.

**Table 9:** Annotated Chitin-degrading Enzymes.

Gene	Blast Prediction	Signal Peptide	GH	CBM	LPXTA Anchor
Bcell_0481	chitinase	yes	GH18		yes
Bcell_2149	chitinase	no	GH18	CBM50 CBM50	no
Bcell_4208	chitinase	no	GH18	CBM50 CBM50 CBM50 CBM50 CBM50 CBM50 CBM50	no
Bcell_3703	chitobiase	no	GH20		no

#### Demonstration of Metabolic and LPXTA enzyme activity

To confirm electronic predictions for metabolic pathways, Bcell monosaccharide and polysaccharide utilization was determined in ATCC 661 Alkaline Bacillus Medium using 2.0 g/l of added carbohydrate as described in Methods. Growth was measured by A_595_ at 24 and 46 hours on duplicate cultures, the complete cultivation experiment was repeated twice, for a total of four replicates. No significant difference in A_595_ was seen between 24 and 46 hr values, indicating completion of growth at 24 hr. ATCC 661 Medium with no added carbohydrate was used as the control; Bcell grew to A_595_∼0.6 on this carbohydrate-free medium. Glucose, xylose, arabinoxylan, glucomannan, β-glucan, galactan, and xyloglucan strongly stimulated growth (A_595_ 2× greater than control). Arabinose, linear arabinan, CMC, chitin, soluble starch, and β-cyclodextrin were less stimulatory, yielding increases of 20% to 50% over control absorbance values. Crystalline cellulose degradation was investigated in a separate series of experiments. Bcell cultures were grown with either 2.0 g/l of added Avicel microcrystalline cellulose or Whatman 1 filter paper. Growth was not significantly enhanced over the control medium, and no change in the amount of cellulose was noted compared to the uninnoculated control, indicating that crystalline cellulose was not a substrate for the organism.

Cultures (5.0 ml) were grown, harvested by centrifugation, washed, and lysed as described in **Methods**. The cell walls and lysates were separated by centrifugation, the cell walls were washed three times with 50 mM Tris-HCl, pH 9.5, and re-suspended in the same buffer. Aliquots of the lysate and cell walls were spotted on ATCC 661 Alkaline Bacillus Medium agar plates containing 100 mg/L of one of the following substrates: 4-methylumbelliferyl-β-D-cellobiopyranoside (MUC) or 4-methylumbelliferyl-β-D-xylopyranoside (MUX) and incubated for 30 minutes at 37°C The cell walls showed strong activity on both MUC and MUX; equivalent aliquots of the lysates showed no activity for either of these two substrate (**Table 10**).

**Table 10:** Activity of Cell Wall Fractions from Cultures Grown on Different Carbohydrates.

Carbohydrate Added	MUCActivityof Cell Walls	MUXActivityof Cell Walls
**None**	+	+
**Glucose**	+++	+++
**Xylose**	+++	+++
**Arabinose**	++	++
**Arabinan**	+++	+++
**Arabinoxylan**	++	+++
**Glucomannan**	+++	+++
**β-glucan**	++	++
**Galactan**	+++	+++
**Xyloglucan**	+	+++
**CMC**	+	++
**Chitin**	++	++
**Soluble Starch**	+	+
**β-cyclodextrin**	+	+

Cultures grown and cell walls prepared and assayed as described in **Methods**. Legend: - no fluorescence; + weak fluorescence, ++ moderate fluorescence, +++ strong fluorescence.

Bcell cultures (50 ml) were grown in media containing galactan, glucomannan, or β-glucan. Cultures were harvested by centrifugation, and the supernatant (S), cell wall (W), and intracellular fractions (I) were recovered as described in **Methods**. To demonstrate that the LPXTA enzymes were crosslinked to the cell wall, an aliquot of each cell wall sample was pelleted by centrifugation and digested with lysozyme. Insoluble material remaining after lysozyme treatment was removed by centrifugation, yielding a lysozyme-solubilized fraction (WL); aliquots of cell walls treated identically, but without lysozyme (WS) served as a control. Aliquots of each sample equivalent to 20 µl of culture were spotted on either ATCC 661 Alkaline Bacillus Medium agar plates containing either fluorescent or chromogenic substrates at 37°C for 30 minutes. The results (**Table 11**) show that the majority of MUC, MUX and X-gal activity is present in the cell wall (W) fraction. Only the culture grown on galactan showed weak activity in the supernatant and intracellular fractions; the other two substrates showed no supernatant or intracellular activity. In all three samples, treatment of the cell walls with lysozyme resulted in solubilization of the enzymatic activity (WL); the solubilization controls (WS) all showed no activity.

**Table 11:** Localization of Enzymatic Activity in Bcell.

Cell Fraction	β-glucan		Glucomannan		Galactan	
	MUC Activity	MUX Activity	MUC Activity	MUX Activity	MUC Activity	MUX Activity
Supernatant	**-**	**-**	**-**	**-**	**++**	**+**
Intracellular Soluble	**-**	**-**	**-**	**-**	**+**	**+**
Intact Cell Wall	**++**	**++**	**+++**	**++**	**+++**	**+++**
Cell Wall Solubilization Control	**-**	**-**	**-**	**-**	**-**	**-**
Lysozyme-Solubilized Cell Wall	**+++**	**++**	**+++**	**++**	**+++**	**+++**

Cultures grown and cell walls prepared and assayed as described in **Methods**. Legend: - no fluorescence; + weak fluorescence, ++ moderate fluorescence, +++ strong fluorescence.

#### Bcell growth is stimulated by fungal cell wall polysaccharides

**Figure 2:**
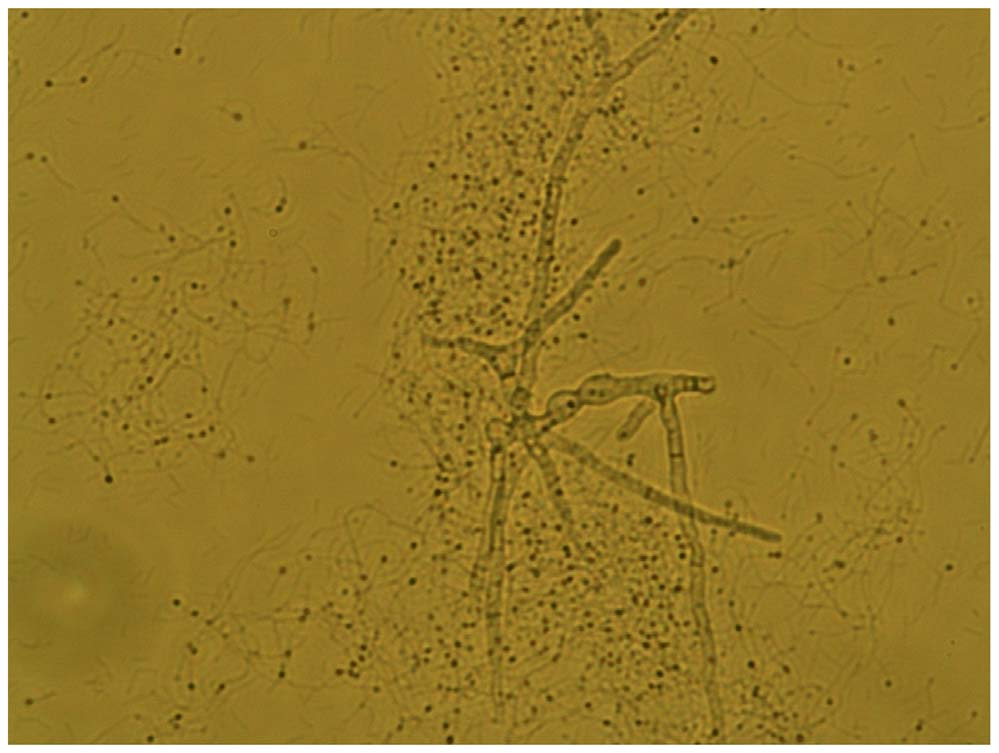
Bcell growth on *A. niger* mycelia. Microscopic images (2000× magnification) of Bcell culture growing on *A. niger* mycelia; same field with transmitted light.

In this work we have demonstrated that Bcell does not degrade and consume crystalline cellulose, after which it was named. Since polysaccharides appear to be the substrate of choice for this organism, a search was conducted to determine a likely substrate for Bcell. Based on the number of chitinases and β-(1,3)-glucanases present in the organism, fungal cell walls seemed a likely source for carbohydrates. To test this hypothesis, *Aspergillus niger* mycelia were grown, harvested, washed, extracted with NaOH, and autoclaved to produce a cell wall fraction. The cell wall fraction was added to ATCC 661 Alkaline Bacillus Medium and autoclaved. Growth was measured by A_595_ at 24 hours on duplicate cultures. The results showed the *A. niger* cell walls stimulated growth better than the most stimulatory carbohydrates such as arabinoxylan, glucomannan, and galactan; growth was 5-fold higher on cultures containing mycelia versus the control (no mycelia) media. Micrographs were taken of the culture after staining with Live-Dead stain. The micrographs (**Figures 2** **and** **3**) show the mycelia (thick, branched rods) covered with adherent bacterial cells (thin, long rods), suggesting that fungal cell wall degradation may also involve attachment of the Bcell cells to the target fungi (fungal mycelia do not stain brightly with the Live-Dead reagent). This attachment to the fungal cell wall would make degradation by the LPXTA enzymes significantly more efficient.

**Figure 3:**
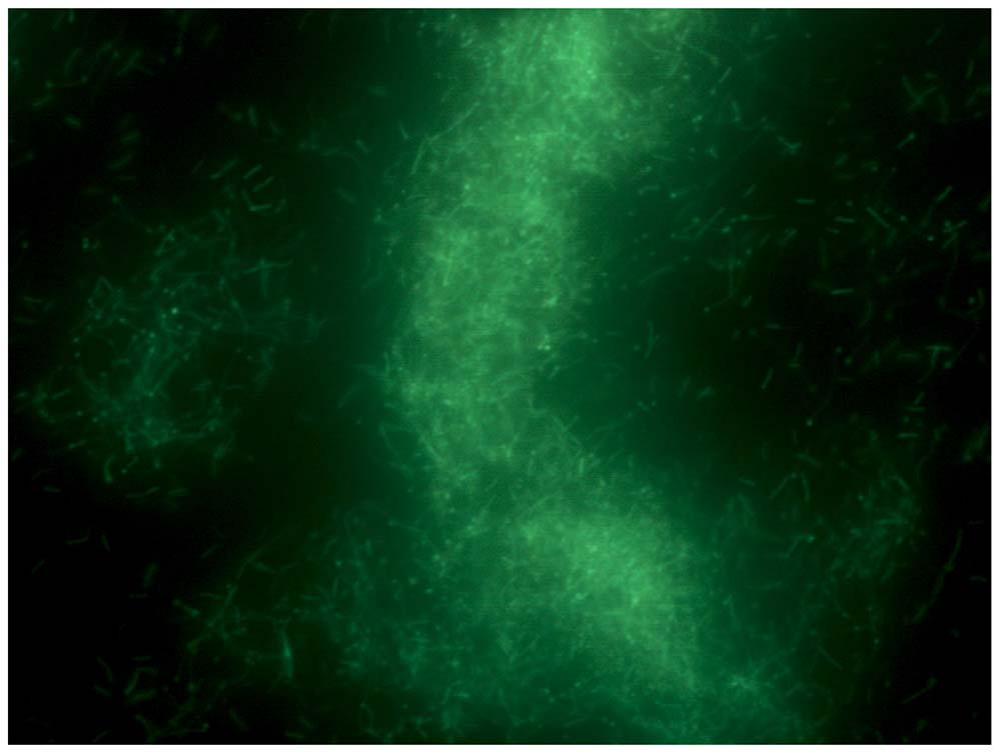
Bcell growth on *A. niger* mycelia. Microscopic images (2000× magnification) of Bcell culture growing on *A. niger* mycelia; same field with dark field epifluorescence.

## Discussion

*Bacillus cellulosilyticus* N-4 DSM 2522 (originally *Bacillus* N-4) was originally isolated from soil in a screen for cellulase producers, and three cellulases genes have been cloned from it [21], [22], [23]. The genome of this organism was sequenced as part of a search for novel, alkaline biomass-degrading enzymes, and revealed Bcell to be an enigmatic organism. Based on the 16S rDNA comparison, Bcell is most closely related to the alkalophile *B. vedderi*, an organism isolated from a bauxite-processing red mud tailing pond [26]. Among organisms with sequenced genomes, it is moderately related to *B. selenitireducens*, a microaerophile isolated from the anoxic mud of Mono Lake in California [46] based on 16S rDNA and whole genome-based comparisons.

The genome of Bcell is a single chromosome of 4.7 M with no plasmids present but three large phage insertions are evident in the genome. The genome contains all genes consistent with its reported carbohydrate metabolism [24] as well as its alkaline and high salt growth. The most unusual feature of the genome is the presence of 27 LPXTA membrane anchor proteins, 17 of which are annotated as involved in polysaccharide degradation. These two values are significantly higher than seen in any other *Bacillus* species. The presence of this large number of LPXTA membrane anchor proteins is also unusual, because Bcell possesses no genes containing either LPXTG or NPXTN sequences. This high number of membrane anchor proteins is only seen in pathogenic Gram-positive organisms such as *L. monocytogenes* or *S. aureus*. Bcell also possesses four sortase D subfamily 4 enzymes that incorporate LPXTA-bearing proteins into the cell wall; three of these are closely related to each other and unique to Bcell. Cell fractionation and enzymatic assay of Bcell cultures show that the majority of polysaccharide degradation is associated with the cell wall LPXTA-enzymes, an unusual feature in Gram-positive aerobes. The evolutionary source of these enzymes is unclear, since no close *Bacillus* or *Geobacillus* relatives of Bcell utilize this unique method of polysaccharide degradation. Furthermore, a BLAST analysis of the LPXTA enzymes of Bcell show they are not closely related to any other enzymes from Gram-positive organisms. Based on genomic comparisons, there is clear evidence that these enzymes are not the result of gene duplication within Bcell, nor were their genes laterally transferred to Bcell from another organism.

Genomic analysis and growth studies both strongly argue against Bcell being a truly cellulolytic organism, in spite of its name [24]. This suggests that a more appropriate name for the organism should be determined based on its true metabolic role in nature. If Bcell does not grow on cellulose and cellulosic materials, what is its preferred substrate? One potential answer is that Bcell degrades fungal mycelia as a preferred carbon source. This is supported by the large number of annotated chitinases and 1,3 β-glucanases present in the genome. The organism could utilize these enzymes to degrade fungal cell walls. Bcell also possesses a wide range of antibiotic resistances, making it resistant to attack. Finally, Bcell possesses two annotated LPXTA adhesion proteins Bcell_0381 and Bcell_2860, as well as several LPXTA hypothetical proteins that may be involved in adhesion to fungi. Future work is needed to determine how Bcell adheres to fungal mycelia and if its isolated enzymes can effectively degrade and consume fungal cell wall components.

While the genomic sequencing did not reveal any new, crystalline cellulose degrading enzymes, the LPXTA-sortase system utilized by Bcell may have application in anchoring cellulases and other biomass-degrading enzymes in other Gram-positive organisms. The possibility that Bcell is capable of degrading fungal cell walls suggest the organism and its enzymes may have potential applications as a biofungicide in clinical and plant protection areas.

## Methods

### Cultivation and Library Construction

*Bacillus cellulosilyticus* DSM 2522 was obtained from the American Type Culture Collection as ATCC 21833 (Designation Bacillus sp. N4 [FERM-P 1141]) and cultivated in ATCC 661 Alkaline Bacillus Medium at 37°C. Cells were harvested in log phase by centrifugation. The cell concentrate was lysed using a combination of SDS and proteinase K, and genomic DNA was purified using phenol/chloroform extraction [47]. The genomic DNA was precipitated, treated with RNase to remove residual contaminating RNA. The genomic DNA was submitted to the Joint Genome Institute of the Department of Energy for whole genome sequencing.

### DNA Sequencing, Assembly, and Annotation

The genome of *Bacillus celluolsilyticus* DSM 2522 was sequenced at the Joint Genome Institute (JGI) using a combination of Illumina [48] and 454 technologies [49]. An Illumina GAii shotgun library with reads of 226 Mb, a 454 Titanium draft library with average read length of 256.2+/−194.9 bp bases, and a paired end 454 library with average insert size of 21 Kb were generated for this genome. All general aspects of library construction and sequencing performed at the JGI can be found at http://www.jgi.doe.gov/. Illumina sequencing results were assembled with VELVET [49], and the consensus sequences were shredded into 1.5 kb overlapped fake reads and assembled together with the 454 sequencing results. Draft assemblies were based on 180.4 Mbp 454 draft data and all of the 454 paired end data. Newbler parameters are “-consed -a 50 -l 350 -g -m -ml 20”. The initial Newbler assembly contained 69 contigs in 5 scaffolds. We converted the initial 454 assembly into a phrap assembly by making fake reads from the consensus, collecting the read pairs in the 454 paired end library. The Phred/Phrap/Consed software package (www.phrap.com) was used for sequence assembly and quality assessment [50], [51], [52] in the following finishing process. After the shotgun stage, reads were assembled with parallel phrap (High Performance Software, LLC). Possible mis-assemblies were corrected with gap resolution (Cliff Han, unpublished), Dupfinisher [53], or sequencing cloned bridging PCR fragments with subcloning or transposon bombing (Epicentre Biotechnologies, Madison, WI). Gaps between contigs were closed by editing in Consed, by PCR and by Bubble PCR primer walks. A total of 627 additional reactions were necessary to close gaps and to raise the quality of the finished sequence. The completed genome was deposited on Dec. 14, 2010 by the US DOE Joint Genome Institute and can be accessed as GenBank: CP002394.1 or NCBI Reference Sequence: NC_014829.1.

Genes were identified using Prodigal [54] as part of the Oak Ridge National Laboratory genome annotation pipeline, followed by a round of manual curation using the JGI GenePRIMP pipeline [55]. The predicted CDSs were translated and used to search the National Center for Biotechnology Information (NCBI) nonredundant database, UniProt, TIGRFam, Pfam, PRIAM, KEGG, COG, and InterPro databases. These data sources were combined to assert a product description for each predicted protein. Non-coding genes and miscellaneous features were predicted using tRNAscan-SE [56], RNAMMer [57], Rfam [58], TMHMM [59], and signalP [42].

### Substrate Evaluation and Cell Wall Preparation

Bcell cultures (5 ml) were grown in Alkaline Bacillus Medium at 37°C containing 2% added carbohydrate. Carbohydrates used were glucose, xylose, arabinoxylan, glucomannan, β-glucan, galactan, xyloglucan, arabinose, linear arabinan, CMC, chitin, soluble starch, and β-cyclodextrin. Galactan, galactomannan, arabinoxylan, arabinan, and β-glucan were obtained from Megazyme International (Wicklow, Ireland). Glucose, xylose, arabinose, lysozyme, CelLytic B reagent and carboxymethyl cellulose (CMC) were purchased from Sigma-Aldrich (St. Louis, MO). Polysaccharides were autoclaved in the medium, while monosaccharides were added aseptically after autoclaving to prevent formation of Maillard reaction products at high pH. Cultures were harvested at 24 hours by centrifugation. Pellets were resuspended in 1.0 ml of 50 mM Tris-HCl, pH 9.5 and pelleted; this was repeated three times. The pellets were then lysed by shaking at 37°C for 30 minutes in 1.0 ml of CelLytic B reagent in 50 mM Tris-HCl, pH 9.5. The pellets containing the cell walls were recovered by centrifugation and resuspended in 1.0 ml of 50 mM Tris-HCl, pH 9.5 and pelleted; this was repeated three times. Finally the pellets were resuspended in 1.0 ml of 50 mM Tris-HCl, pH 9.5.

Larger volume Bcell cultures (100 ml) were grown in Alkaline Bacillus Medium at 37°C containing 2% added glucomannan, β-glucan, or galactan and fractionated as described above. For lysozyme treatment, 1.0 ml of cell walls was pelleted by centrifugation, and resuspended in 0.1 ml 50 mM Tris-HCl, pH 8.0; 2.0 µl of 10/mg ml lysozyme in 100 mM Tris-HCl, pH 8.0 was added and incubated overnight at 37°C. A control was used in which 2.0 µl of 100 mM Tris-HCl, pH 8.0 was added instead of lysozyme. Solublized cell wall components were recovered by centrifugation.

### Enzymatic Analysis

Qualitative enzyme activity assays were conducted at 37°C on agar plates containing 100 mg/l of substrate. *exo*-Glucanase, *exo*-xylanase, and β-galactosidase were determined using either YT or ATCC 661 Alkaline Bacillus Medium plates containing 100 mg/l of 4-methylumbelliferyl-β-D-cellobiopyranoside (MUC) (cellulase and *beta*-glucanase) or 4-methylumbelliferyl-β-D-xylopyranoside (MUX) (*beta*-xylosidase), MUC and MUX were obtained from Research Products International Corp. (Mt. Prospect, IL).

### Fungal Growth and Lysis

An *Aspergillus niger* culture (1000 ml) was grown for 7 days at 30°C in MRS medium (Thermo-Fisher (Fitchburg, WI)) containing 4% added glucose. The mycelia were recovered by centrifugation, washed with deionized water and recovered by centrifugation. This was repeated for a total of three washes. The mycelia were then suspended in 200 ml of 1.0 M NaOH and stirred for 2 hours and the extracted mycelia were recovered by centrifugation and washed twice with deionized water. Alkaline Bacillus Medium containing 5% added wet weight extracted mycelia was used for growth experiments.

For microscopy experiments, 3 µl of each stain in the Live-Dead® BacLight™ Bacterial Viability Kit (Invitrogen Molecular Probes (Eugene, OR)) was added to 1.0 ml of deionized sterile water. The diluted stain (20 µl) was added to 100 µl of culture, and incubated for 10 minutes at room temperature in the dark before microscopic observation. Samples were observed using a Nikon Eclipse TE2000-S epifluorescence microscope (excitation 450–490 nm, emission >500 nm) and pictures were taken using a Diagnostics Instruments Model 11.2 Color Mosaic camera and processed using Diagnostics Instruments Spot Advanced software.
